# Toward the Role of Teacher Empathy in Students’ Engagement in English Language Classes

**DOI:** 10.3389/fpsyg.2022.880935

**Published:** 2022-06-02

**Authors:** Zhichao Zhang

**Affiliations:** College of Foreign Languages, Henan Institute of Science and Technology, Xinxiang, China

**Keywords:** teacher empathy, teacher-student rapport, positive environment, learner engagement, kindness

## Abstract

This paper aimed at examining the related studies on the relationship between teacher empathy and learner engagement in English as a Foreign Language (EFL) learning contexts. The positive and significant relationship between teacher empathy and learner engagement has been verified in the literature. Studies have shown the positive influence of teacher-learner rapport on learner engagement. Moreover, studies indicated that teacher empathy leads to learner self-confidence in educational contexts. The literature review has also shown that teachers’ provision of a positive environment through empathy, sympathy, and kindness can trigger learner motivation and engagement. Moreover, teachers’ empathy can reduce the stress level which, in turn, positively affects learners’ engagement level. Eventually, the pedagogical implications to engage learners and teachers in academic contexts have been provided. However, some suggestions have been offered to expand the insights over the associations between positive psychological constructs and teachers’ emotions.

## Introduction

Emotions have been considered important components that affect instructors’ behaviors in educational contexts. In effect, many studies have tended to explore teacher emotions and their influences on learners’ academic achievement and engagement (Xie and Derakhshan, 2021). Language teachers are required not only to have language proficiency, the capability of designing approaches and methods, the ability to assess and evaluate learners, and the aptitude to use numerous instruction aids but also to build their relationship with learners through considering their negative and positive affectivities (Liu, 2016). When learners feel that they are not connected to their instructors, their insights toward the efficiency of education are reduced (Moore and Kearsley, 2004). Moreover, learners are inclined to engage more in classroom contexts if they have a positive and close relationship with their instructor (Richardson et al., 2012). Therefore teacher-learner rapport, particularly teacher empathy, can trigger learners to engage more in classroom contexts. On the other hand, engagement, as the positive psychology construct, has drawn the attention of positive psychologists, since they try to enhance learning outcomes and foster learning contexts (Wang et al., 2021). Seligman (2011) developed a positive psychology construct, and he called it five-dimensional PERMA, including “positive emotion (P), engagement (E), relationships (R), meaning (M), and accomplishment (A)” (p. 12). He asserted that the purpose of positive psychology is to prosper these dimensions of positive psychologists. However, investigators tended to ponder into the positive emotional constructs with the aim of helping learners to process language better in their minds (Fang and Tang, 2021). This paper tries to investigate the related literature about the role of teacher empathy in learner engagement. The significance of this study is to raise teacher awareness toward their positive and negative behaviors and their effects on learners’ willingness to involve in educational contexts.

## Literature Review

### The Concept of Empathy

Eisenberg et al. (2014) defined empathy as individuals’ capability to appreciate and share others’ negative and positive emotions. Weisz and Cikara (2021) also pointed out that empathy denotes the realization of the opinions, and emotional states of individuals, and attending to their wellbeing. Amicucci et al. (2021) also stated that empathy can be described as a significant component of interpersonal behaviors. Baron-Cohen and Wheelwright (2004) expressed that empathic individuals show their effort and persistence to spot others’ thoughts and react to them. They maintained that empathy, as an inner feeling, helps individuals predict other people’s behaviors. In a study, Cialdini et al. (1997) asserted that empathy consists of definite capabilities instead of attitudes alone. Mercer and Reynolds (2002) regarded empathy as a multi-dimensional construct with ethical, cognitive, emotional, and interactional constituents and has been theorized in numerous fields. Studies have presented two key categories of empathy: cognitive and affective (Stojiljković et al., 2012). Cognitive empathy is concerned with understanding others’ emotional involvements (Blair, 2006). On the other hand, affective empathy refers to sharing other individuals’ emotional experiences (Reniers et al., 2011). Studies have shown that cognitive and affective empathy are different, but they are significantly correlated (Smith and Rose, 2011). Tusche et al. (2016) stated that autistic individuals, for example, are inclined to have cognitively, not affectively, deficient in empathy; while psychopathic individuals have a deficiency in affective empathy. They also argued that various brain parts belong to cognitive and affective empathy. Eisenberg et al. (2014) stated that empathy is commonly regarded as a helpful social-emotional aptitude for group interactions. Investigations have shown that individuals may feel pain or happiness when perceiving pain or happiness Studies have shown that empathy can predict individuals’ psychological wellbeing in educational contexts (Vinayak and Judge, 2018; Wang and Guan, 2020). Empathy is significantly correlated with psychological wellbeing, since taking others’ viewpoints in to account, specifies doing away with egotistic perspective of one’s own self, and then, helps in decrease of selfish and imprudent behavior, which in turn contributes toward enhanced wellbeing (Gazzaniga, 2008). Rajabi and Ghezelsefloo (2020) also asserted that English as a Foreign Language (EFL) teachers’ self-compassion in others, leading to high levels of empathy, kindness, and compassion (Gao et al., 2016). Therefore teachers’ empathy can decrease theirs stress level and increase their psychological wellbeing. Moreover, the positive correlation between self-compassion and psychological wellbeing has been verified (Wang et al., 2022).

#### Teacher Empathy

Empathy is regarded as an important quality for instructors, medical doctors and social workers, and those interacting with individuals (Stojiljković et al., 2012). Since cognitive and affective categories of empathy are significant for interactive operations, they are useful for occupations that need emotional support from society. Although many studies have been done on the empathy of psychoanalysts and physicians, few investigations highlighted teacher empathy (Hen, 2010). Studies have revealed that empathy plays an important role in the development of teachers and learners in terms of ethical, communal, and educational issues (Arghode et al., 2013). Concerning the educational contexts, Rogers (1995) stated that “when a teacher has the ability to understand the student’s reactions from the inside, has a sensitive awareness of the process of how education and learning seem to the student…then the likelihood of learning is significantly increased” (p. 157). Tettegah and Anderson (2007) defined teacher empathy as an aptitude to communicate with learners’ concerns and understand their concerns and perceive the situations from learners’ points of view. Moreover, Cooper (2010) found out that teachers can convert a context into a place for education, through satisfying the demands of learners and responding to their apprehensions. Ikiz (2009), in an investigation, found out that empathic teachers control learners’ violence, increase their psychological wellbeing, and decrease their violent actions. Serbati et al. (2020), in a study about exceptional instructors, highlighted the importance of teacher empathy, teacher motivation, and learner cooperation as valuable factors for teaching exceptional learners. Teacher empathy is affected by numerous variables such as gender (Pidbutska et al., 2021), and attitude (Parchomiuk, 2019). Klassen et al. (2017) found the relationship between personality traits like teacher agreeableness with teacher empathy which enhances teaching effectively. Wink et al. (2021) studied the relationship between teachers’ cognitive empathy, mindsets, and job burnout. They found out that cognitively empathic teachers tend to have positive mindsets about learners’ performance, and they have an ability to manage learners’ problematic behaviors. They also found out that teachers with higher levels of cognitive empathy are inclined to use problem−solving strategies, and they have lower levels of job burnout in educational contexts. Trying to inspect the patterns of teacher empathy, Zohoorian and Faravani (2021) found fantasy, personal apprehension, teacher engagement, autonomy, and different personality traits as the key elements influencing teacher empathy. Hassanpour Souderjani et al. (2021), in another study, highlighted the importance of life satisfaction in teacher empathy, and they implicated that providing appropriate conditions can be helpful to increase teacher empathy. Zhu et al. (2019) also found out that teachers’ professional identity positively influences teacher empathy. The significant correlation between EFL teachers’ emotional intelligence and teacher empathy was approved in the study conducted by Salem and Tabatabaei (2015). Csaszar et al. (2018) also found teacher compassion as a mediating factor in the relationship between teacher empathy and apprehension.

Cooper (2004) asserted that teachers with high levels of empathy help learners to augment their self-efficacy and motivation. Hen (2010) found out that self-efficient instructors have significantly high levels of empathy and optimism toward learners with special needs in a mainstream classroom. Lam et al. (2011) also found a significant positive correlation between empathy and teacher-learner rapport among science teachers in educational contexts. Gandhi et al. (2021) argued that teacher belief can be considered an important component that affects teacher empathy and motivation. Weisz et al. (2021) also found that teachers who believed in the changeability of empathy showed more minor offensive behaviors and great empathy. Goroshit and Hen’s (2016) study revealed that instructors’ self-efficacy and emotional self-efficacy are significantly related to teacher empathy. They found out that teacher self-belief plays a mediating variable in the association between teacher self-efficacy and teacher empathy.

### Learner Engagement

According to Lamborn et al. (1992), engagement is described as “learners’ psychological effort and investment toward learning, understanding, or mastering the skills, crafts, or knowledge that the coursework is intended to promote” (p. 13). However, Skinner et al. (2009) defined learner engagement as “the quality and quantity of students’ participation or connection with the educational endeavor and hence with activities, values, individuals, aims, and place that comprise it” (p. 495). Lei et al. (2018) pointed out that learners’ engagement denotes the time that learners spend efficiently doing educational tasks and activities. Chang et al. (2016) asserted that learner engagement is described as the amount of learner involvement in educational contexts such as face-to-face or face-to-screen environments and the amount of energy they use on educational projects. Mostly, in language education, investigating engagement can elucidate learners’ contemplation, beliefs, and emotions in instructional contexts (Oga-Baldwin, 2019). Hiver et al. (2021) also argued that the learner engagement construct is multifaceted and includes numerous features such as emotional, cognitive, and behavioral aspects. They asserted that learners can, not only physically but also cognitively, involve in the classroom by getting done a language learning assignment. They maintained that these features interact to determine learners’ optimism toward the learning process. Dincer et al. (2019) stated that some activities such as doing tasks, classroom participation, and interacting with teachers in terms of asking and answering questions are related to behavioral engagement. Mercer (2019) stated that the behavioral dimension of engagement distinguishes engagement from motivation. Dincer et al. (2019) also defined emotional engagement as learners’ emotional reactions in classroom contexts. Moreover, they described cognitive engagement as learners’ tendency to use complicated learning strategies instead of simple strategies. Reschly et al. (2020) asserted that behavioral engagement is significantly correlated with cognitive and affective engagement. Reeve (2013) expanded the domain of engagement and introduced agentic engagement as the fourth feature of this construct. According to Reeve and Tseng (2011), agentic engagement refers to “students’ constructive contribution into the flow of the instruction they receive” (p. 258). Reeve (2013) also stated that agentic engagement is regarded as “proactive, intentional, collaborative, and constructive student-initiated pathway to greater achievement” (p. 579). Guo (2021), in studying Chinese contexts, found out that Chinese EFL learners are cognitively, behaviorally, and emotionally involved in educational contexts. However, they did not involve agentically in the educational contexts.

Some studies have been done on the relationship between engagement and other positive psychological constructs such as grit, wellbeing, foreign language enjoyment, hope, and other emotions like motivation. Yang (2021) showed a significant correlation between EFL learners’ grit, wellbeing, and engagement. It can be concluded from his study that EFL gritty learners with high levels of engagement tend to have higher levels of wellbeing. Robinson (2015) investigated the relationship between engagement and grit among nursing students. Her study revealed that course engagement is significantly associated with learners’ grit. Moreover, she found out that persistence of effort is significantly correlated with learners’ behavioral engagement. O’Neal et al. (2019) compared elementary learners’ grit with their emotional engagement. They operationalized emotional engagement as learning enjoyment. They reasoned that motivation, as an important mediator, plays a key role in the significant relationship between grit and emotional engagement. Derakhshan (2021) also argued that learners with high levels of grittiness in learning a language, usually evaluate their own activities, and foster their academic engagement and enjoyment. Gallagher et al. (2017) highlighted the role of hope in increasing learners’ academic success. He also mentioned the positive correlation between hope and learners’ engagement in English courses. Reilly and Sánchez Rosas (2019) argued that language learning enjoyment and hope are significantly correlated, and they mentioned that higher proficient experience learners have higher levels of engagement, enjoyment, and hope during learning. In contrast, low proficient learners have more apprehension and hopelessness. Eccles (2016) argued that learner academic engagement has a positive and significant relationship with academic achievement and resilience. Oga-Baldwin and Nakata (2017) stated that engagement is strongly correlated with intrinsic motivation; however, they found out that engagement is negatively correlated with extrinsic motivation. Their study also revealed that male learners tend to be less engaged in the classroom, and they have lower intrinsic motivation. Ryan and Deci (2017) also found out that engagement increases a self-decided motivational orientation. Peng (2021) considered the learner motivation a precondition for learner engagement and academic achievement. Lin (2012) also pointed out that learners’ cultural and educational backgrounds, and teacher attitudes toward the learner can affect their academic engagement and motivation.

Ghelichli et al. (2022) found significant correlations between language learning motivation and each aspect of learner engagement. Their study also revealed the strongest significant correlation between cognitive engagement and language learning motivation. In another study on the relationship between negative emotions and learner engagement, Zhao et al.’s (2021) study revealed that learner academic engagement is significantly correlated with learner mindset. They also mentioned that stress, as a negative emotion, mediates the correlation between learning engagement and growth mindset.

Teachers’ use of methodologies is also effective in academic engagement. Guilloteaux (2016) asserted that learners’ engagement is affected by their background level, task type, task difficulty, instructors’ methodology, motivation, and teaching style. Hung (2015) argued that memorization and rote learning, as two outdated teaching approaches, significantly predict learners’ disengagement. He suggested flipped instruction for solving disengagement. Using the teaching style of Grasha (1996) and Shaaria et al. (2014) found a positive correlation between teaching styles and learners’ academic engagement. In another study, Ghaznavi et al. (2021) inspected the role of teaching multiple intelligences in learner engagement, and they found out that this type of teaching approach can enhance learner multiple intelligence, which in turn, develops learner academic engagement.

Recently, learners’ engagement has been proved as an important factor in solving educational problems, including low achievement, high dropout proportions, and high levels of learners’ weariness and violence (Boekaerts, 2016). Using self-determination theory, Chen et al. (2021) argued that the relationship between context-based characteristics and learners’ mental requirements influences learner engagement in an educational context. Hospel and Galand (2016) also stated that the instructor plays a key role in facilitating the meeting of learners’ needs and engagement through providing autonomy support. They argued that the autonomy support that instructors provide increases learners’ engagement. Reeve and Shin (2019) also found out that instructors’ autonomy-support is regarded as a significant predictor of learners’ engagement. Noels K. et al. (2019) also approved the positive and significant correlation between engagement, motivation, autonomy, and competency.

### The Role of Teacher Empathy in Learner Engagement

Some important moral values and merits, including teacher empathy, sympathy, tolerance, teacher kindness, and justice as the components of teacher-learner relationships, are incorporated in the instructional frameworks (Campbell, 2003). Roorda et al. (2017) stated that positive and emotional relationships between teachers and learners are influential in learners’ academic achievement. It has also showed that positive teacher-learner rapport can inspire learning motivation and engagement (Wang and Guan, 2020; Xie and Derakhshan, 2021; Wang et al., 2022). Vandenbroucke et al.’s (2018) study revealed that teacher-learner rapport positively impacts learners’ cognitive skills. Zhou (2021) aimed at studying teacher-learner rapport, and its positive effect on learner engagement in educational environments. The positive influence of teacher-learner positive relationships on learner engagement was elucidated in his study. He argued that establishing rapport and empathy in educational contexts requires teachers’ devotion to learners’ wellbeing, and their permission to learners for sharing and easily articulate their emotions concerning instruction and their life. Schutz and DeCuir (2002) stated that empathic instructors intensify learners’ willingness to participate in educational contexts, and improve their self-reliance in the school environment. Varga (2017) also found out that learners’ desire to participate in a classroom context is positively affected by their behavior with instructors. Nathan (2018) suggested that teachers with an aptitude for creating a positive relationship with their learners and understanding their problems can efficiently enhance learners’ willingness to participate in learning and their achievement, which in turn, contributes to their amplified self-sufficiency.

Constructing a positive emotional relationship with learners in educational contexts is crucial for educators, since their positive emotional behaviors like compassion, empathy, sympathy can engage learners in academic contexts to achieve their purposes (Frisby et al., 2016; Wang, 2017; Wang et al., 2022). Cooper (2002) indicated that positive personal interaction, such as teacher empathy, is significantly correlated with teaching quality, learning achievement, learner engagement in educational contexts. Arnold (2009) pointed out that “an affectively positive environment puts the brain in the optimal state for learning” (p. 146). He maintained that empathy produced in positive teacher-learner rapport can significantly diminish anxiety and result in improved engagement. Rogers et al. (2014) also stated that instructors act as facilitator models and they should use empathy, truthfulness, and respect during interaction with learners, and they should build up emotional safety, autonomy, engagement, and inquisitiveness which trigger learners to be deeply involved in classroom contexts. Hashim et al. (2014) verified the significant relationship between teacher-learner rapport and learners’ engagement. They argued that English teachers’ love and empathy toward learners and their wellbeing can increase learners’ tendency to participate in educational contexts. Similarly, Wang et al. (2022) also stated that instructors should incorporate positive emotional behaviors, such as honesty, appreciation, empathy, and sensitivity to learners’ requirements, in their instructional practice, since these behaviors fundamentally generate motivation and engagement among learners. Bullough (2019) also stated that teacher empathy not only increases learner engagement, but also contributes to teachers attaining social justice across different contexts.

Mercer and Dörnyei (2020) asserted that learner engagement is instructor-dependent and it is a dynamic construct. They argued that instructors can take important actions to enhance EFL learners’ commitment. Consequently, they can show empathy, compassion, kindness, and numerous kinds of support to improve learners’ behavioral and emotional engagement. Khan and Armstrong (2019) argued that the enhancement of compassion, empathy, and sympathy among teachers are the critical issues to be considered in order to develop learner engagement and create pleasant performance among learners. In another study, Sadoughi and Hejazi (2021) demonstrated that teacher support is directly related to academic engagement. They argued that learners, who feel empathy, affection, and support from their instructors, usually persist in education, enhance self-confidence, and enhance their willingness toward the educational contexts. Derakhshan (2021) indicated that empathic instructors play ethical roles for their learners by helping them engage in interactions with their peers. He argued that such interaction fosters the educational quality and helps learners enhance their positive behaviors. Teo et al. (2022) showed that English teachers’ close relationship with learners has significantly correlated with learners’ involvement. Their study indicated that teacher immediacy enhances learner engagement in the game-based flipped ESP educational contexts.

## Implications and Suggestions for Further Research

This paper investigated the role of teacher empathy in learners’ willingness to participate in class. This paper enhances the educational knowledge of investigators who are interested in learners’ and teachers’ emotions. Regarding the related literature about the positive role of learner-teacher rapport in learner engagement, it is worth noting that learners should be helped to manage, adapt, and normalize their emotions in educational environments. Learners can control their affections to increase their enjoyment, and this issue can inspire teachers to take into account learner affections in practical classroom contexts. The development of empathy and engagement among learners to engage enthusiastically in classrooms can be considered one of the pedagogical implications of this paper. Learners can use opportunities that teachers provide to express their thoughts and feelings, which can reduce their stress in front of other learners and encourage them to be accountable in their learning. This study can also inspire instructors, teacher educators, and policymakers to brood over EFL learners’ behaviors and their academic engagement. Also, being aware of learners’ personality traits may encourage teachers to do their best to bring passion to EFL learning contexts. Therefore, L2 instructors are required to talk to learners about their internal and external motivation and ask their problems to improve learners’ points of view and motivation to engage in educational contexts. They can increase other positive emotions such as foreign language enjoyment, pride, and hope, and reduce negative feelings such as communication apprehension, disengagement, etc., in their classes.

Moreover, teacher-learner rapport and understanding learners’ needs should be one of the fundamentals of instruction in language education contexts to achieve instructional outcomes. To do so, teachers need to reconstruct and moderate the instructional materials. They can find authentic and interactive materials to increase learners’ positive attitudes and engagement for developing their outcomes. This can diminish learners’ cognitive load and despair, and arouse their enthusiasm and their concentration on language learning contexts. Teacher-learner rapport may persuade teachers to change their approach to teaching by incorporating positive emotions in their methodology to inspire learners to engage in learning. Therefore, they can offer warming-up activities for learning contexts and brainstorm learners to increase learner engagement. The demanding projects, lectures, conferences, and workshops may discourage learners from participating in academic contexts and put additional strain on learners. In order to motivate and engage learners, familiarizing learners with questions of the tests can be helpful, and teachers can change their assessment approach in classrooms. Teachers can manage the time of classrooms regularly, and learner engagement to arouse motivation. Through asking and answering questions, learners can be more engaged during the course and learn information efficiently. Providing a competitive educational context through quizzes boosts learner engagement. Unplanned quizzes are primarily significant for exciting, less engaged, and unprepared learners. Collaboration is another way for teachers to increase learner engagement. Learners are inclined to engage in classrooms when they cooperate on class projects.

Instructing with videos, listening to the learners’ comments and problems and giving feedback can arouse learner engagement. Furthermore, teacher educators can exploit the related studies through consideration of the instructors’ interpersonal behaviors, empathy, and their rapport with the learners. They can hold workshops and provide some strategies to improve teacher empathy. They can also emphasize modeling empathy, taking actions and improving listening, and trying not to interrupt learners while they are speaking. Teacher educators can assess and validate the effectiveness of instructors’ methods on learners’ engagement in EFL contexts. This paper recommends that teacher educators should have a positive view toward teachers and learners, and they should provide wellorganized and inspiring teaching methodologies which can construct a motivation for language learning and engagement in the classroom. Teacher educators should provide elbow support to enhance pedagogical skills in online instruction. They should develop confidence and competence among in-service teachers to entice learners’ interests and engage them in the learning process. Policymakers can develop engagement programs that help learners decrease their communication apprehension, and amplify their academic engagement. They can positively support learners and make a context in which learners can take part in positive behaviors. They can hold academic workshops to help teachers increase engagement among learners. They can provide interesting facilities and positive learning contexts for increasing positive behaviors and motivations among learners. The importance of engagement and empathy may make consultants expand their agendas to diagnose learners’ disengagement reasons, and the obstacles they cope with within language learning.

Future studies may consist of investigating the influence of other individual variables, including learner’s extroversion, introversion, and many others on their different types of engagement. Teachers’ negative emotions such as boredom, burnout, apprehension, and exhaustion and their role in learner engagement should be examined. Other studies can be done to investigate the effect of teacher sympathy and love on learners’ academic engagement. Longitudinal studies are required to shed light on the intrapersonal and interpersonal emotions in language learning. Future studies can investigate the effects of teacher-student rapport, particularly teacher empathy, on learners’ working memory and emotional intelligence. Moreover, the effects of teacher empathy on the enhancement of language skills ought to be considered in detail. Further studies are needed to determine teachers’ empathy, sympathy, and kindness in traditional and digital contexts to illuminate how these contexts may affect learners’ emotions. Besides, further research can be done to investigate the gender effect on teacher empathy in language learning contexts. Finally, future studies should pinpoint the relationship between EFL learners’ emotional intelligence and disengagement in foreign language contexts.

## Ethics Statement

The studies involving human participants were reviewed and approved by the Henan Institute of Science and Technology Academic Ethics Committee. The patients/participants provided their written informed consent to participate in this study.

## Author Contributions

The author confirms being the sole contributor of this work and has approved it for publication.

## Conflict of Interest

The author declares that the research was conducted in the absence of any commercial or financial relationships that could be construed as a potential conflict of interest.

## Publisher’s Note

All claims expressed in this article are solely those of the authors and do not necessarily represent those of their affiliated organizations, or those of the publisher, the editors and the reviewers. Any product that may be evaluated in this article, or claim that may be made by its manufacturer, is not guaranteed or endorsed by the publisher.
